# Metabolic stress in patients with acute severe ulcerative colitis - a single-center cohort study

**DOI:** 10.3389/fendo.2024.1395686

**Published:** 2024-11-08

**Authors:** Mathias Redsted, Magnus Grønhøj, Louise Dalsgaard Brøchner, Janne Fassov, Mads Vandsted Svart, Jens Rikardt Andersen, Christian Lodberg Hvas

**Affiliations:** ^1^ Department of Nutrition, Exercise and Sports, University of Copenhagen, Copenhagen, Denmark; ^2^ Department of Hepatology and Gastroenterology, Aarhus University Hospital, Aarhus, Denmark; ^3^ Steno Diabetes Center Aarhus, Aarhus University Hospital, Aarhus, Denmark; ^4^ Department of Internal Medicine and Endocrinology, Aarhus University Hospital, Aarhus, Denmark

**Keywords:** colitis, ulcerative, basal metabolism, metabolic stress, insulin resistance, prednisolone

## Abstract

**Background and aims:**

Acute severe ulcerative colitis (ASUC) is characterized by systemic inflammation, which may initiate an acute-phase response leading to hypercatabolism. Patients with ASUC are usually treated with high-dose steroids that may further accelerate the metabolic response and lead to hyperglycemia and insulin resistance. Nevertheless, the degree of synergy between inflammation and steroid treatment and their influence on the insulin resistance remains unknown. We aimed to measure the degree of metabolic stress including insulin resistance in patients with ASUC during admission and three weeks after discharge.

**Methods:**

This single-center cohort study was conducted in adult patients with ASUC, defined and assessed by Truelove and Witt’s criteria. Indirect calorimetry, bioelectrical impedance analysis, and the Homeostatic Model Assessment for Insulin Resistance (HOMA-IR) were applied at baseline and at follow-up three weeks after discharge.

**Results:**

Among the 22 patients admitted during the project period, 15 provided consent for participation in the study. Median C-reactive protein at inclusion was 37.6 [4; 154.7]. Both median HOMA-IR and fasting plasma glucose were markedly increased at inclusion (median 8.6 [3.8; 14.1] and 7.1 [6; 8.7], respectively), and both had decreased significantly three weeks after discharge (p=0.0036 and p=0.0039, respectively). No significant differences were observed in resting energy expenditure or anthropometric measurements from baseline to follow-up.

**Conclusion:**

Patients with ASUC presented with marked insulin resistance, indicating that the days following admission and high-dose steroid treatment are particularly vulnerable. Despite improvement at three-week follow-up, patients still exhibited insulin resistance compared with relevant control groups.

**Clinical trial registration:**

ClinicalTrials.gov, identifier NCT0527183.

## 1 Introduction

Ulcerative colitis (UC), the most common chronic inflammatory bowel disease (IBD), causes superficial ulcerations in the colonic mucosa. The exact etiology behind UC is still unknown but is assumed to be due to the interaction between several risk factors, especially genetics and environment (1). In UC, innate and adaptive immune responses are central to the pathogenesis, in which the innate lymphoid cells are implicated in chronic intestinal inflammation Defects related to the adaptive immune system have also been observed in UC, e.g. an abnormal T-cell representation with increased occurrence of especially T-helper cells 2 & 17 (2, 3). This condition leads to an increased activation of the immune system via the secretion of cytokines and macrophages. The occurrence of proinflammatory cytokines is known to be upregulated in UC, especially Tumor necrosis factor alpha (TNF-α) and interleukin (IL)-5, IL-6, IL-12 and IL-17 (4). Thus, the disease follows a patient-specific pattern with intermittent remission periods and a dysregulated immune response with release of cytokines and catabolic hormones, including cortisol, glucagon, and catecholamines (5). The cascade reaction between an increased production of cytokines and catabolic hormones contributes to the breakdown of peripheral tissue, as an acute response to the liver’s accumulated substrate needs. This reaction will happen via up-regulation of proteolysis from fat-free mass (FFM) and lipolysis in adipose tissue, as well as reduced protein synthesis, which correlates with the degree of the stress response. This may potentially initiate a stress-metabolic response, resulting in hypercatabolism and insulin resistance. These mediators influence several metabolic processes, including glycogenolysis and gluconeogenesis (4). Acute severe ulcerative colitis (ASUC) is the most grave presentation of a UC flare, affecting approximately 20% of patients and usually requiring hospitalization and treatment with intravenous high-dose steroids (0.75-1 mg/kg/day) (6, 7).

During an acute-phase response, hyperglycemia may occur as a cause of an increased release of glucose and/or reduced glucose uptake in insulin-dependent tissues (skeletal muscle and adipose tissue). This is associated with prolonged hospital stays, higher risk of infections, delayed wound healing, and increased hospital mortality rates, among others (8, 9).

In addition to metabolic stress, treatment with systemic corticosteroids potentially contributes to peripheral insulin resistance and poor glycemic control. Treatment with high-dose steroids may lead to steroid-induced diabetes mellitus, presumably depending on the treatment duration and the absolute dose (10). The aim of this study was to observe and quantify the degree of stress metabolism and insulin resistance in patients with ASUC during admission compared with the subsequent remission period.

## 2 Materials and methods

### 2.1 Study design and population

This was a single-center cohort study, conducted at a referral center for gastroenterology in Aarhus, Denmark, from February to June 2022. UC patients >18 years of age were eligible when defined as having ASUC based on Truelove and Witt’s clinical and paraclinical criteria. Patients who were pregnant and/or lactating and patients with type 1 or type 2 diabetes were excluded. Before patient enrollment, a specialist physician from the department confirmed the UC diagnosis and assessed clinical criteria according to Truelove and Witt (11). Applicable participants were then invited to consent for enrollment in the study by the study staff. Participants were examined at baseline (two-three days after hospitalization/admission) and at follow-up three weeks after discharge. Disease remission was defined as C-reactive protein (CRP) <4.0 mg/L and Simple Clinical Colitis Activity Index (SCCAI) <5. During hospitalization, all patients with ASUC received high-dose intravenous steroid treatment 40 mg twice daily of Solu-Medrol, Pfizer (Methylprednisolone) at a standard five-day duration before changing to an oral, tapered 10-week steroid regimen, starting at 50 mg prednisolone daily.

Data on Homeostatic Model Assessment of Insulin Resistance (HOMA-IR), HOMA-IR, CRP, and Bioelectrical Impedance Analysis (BIA) were collected in two reference groups. The first group included inpatients admitted to the department with other hepato-gastroenterological diseases than UC (seven patients with liver diseases, four patients with gallbladder infection and one patient with clostridium infection) and with active inflammation defined as a CRP above 25 mg/L. The second group included outpatients with UC in remission receiving biological treatment in the outpatient clinic. Disease remission was defined by a SCCAI <5 and a CRP below the detection limit (<4.0 mg/L).

### 2.2 Measurements and statistical analyses

Investigations were made at baseline and at follow-up in the morning after an overnight fast of at least six hours. The investigations consisted of blood samples, BIA (Seca mBCA 515, Hamburg, Germany), and indirect calorimetry (IC) measurements (COSMED Q-NRG, Rome, Italy). Furthermore, the patient’s height, weight, Body Mass Index, skeletal muscle mass, fat mass, fat free mass, phase angle, resting energy expenditure (REE), and respiratory quotient (RQ) were measured and calculated.

The blood samples included CRP, hemoglobin, fasting plasma glucose, and fasting plasma insulin. All venous blood samples were analyzed immediately after collection by routine in-house analytical methods. Insulin resistance was assessed using HOMA-IR based on fasting plasma glucose and insulin (12).

Prior to measurement of BIA and IC, the patients were instructed to wear light clothing and refrain from any physical activity for a minimum of one hour. The IC was measured using a canopy, and the patients were laying in a hospital bed in a supine and relaxed position. IC was applied after enrollment of the fourth study patient.

HOMA-IR was used to detect insulin sensitivity and estimated based on the following formula (12):


$$HOMA-IR=\frac{fasting\;p-glucose\left(mmol/l\right)x\left(p-insulin\;(mIU/l\right)}{22.5}$$

A HOMA-IR of 1 indicated normal insulin sensitivity, whereas values >2.0 indicated varying degrees of insulin resistance (13).

Data were analyzed using the Wilcoxon signed-rank test for paired observations according to our study hypothesis. Spearman’s correlation was used to examine covariation between monotonic variables. The Mann-Whitney test was used to compare the HOMA-IR between the study patients and reference groups. Continuous data were reported as median [range]. Statistical significance was set at a p-value <0.05. In case of missing data, pairwise deletion was applied. All data were analyzed using GraphPad Prism (version 9.4.0) (GraphPad Software, La Jolla California USA, www.graphpad.com). Data were managed in REDCap, hosted by Aarhus University (redcap.au.dk).

## 3 Results

### 3.1 Demographic data

**Figure 1:**
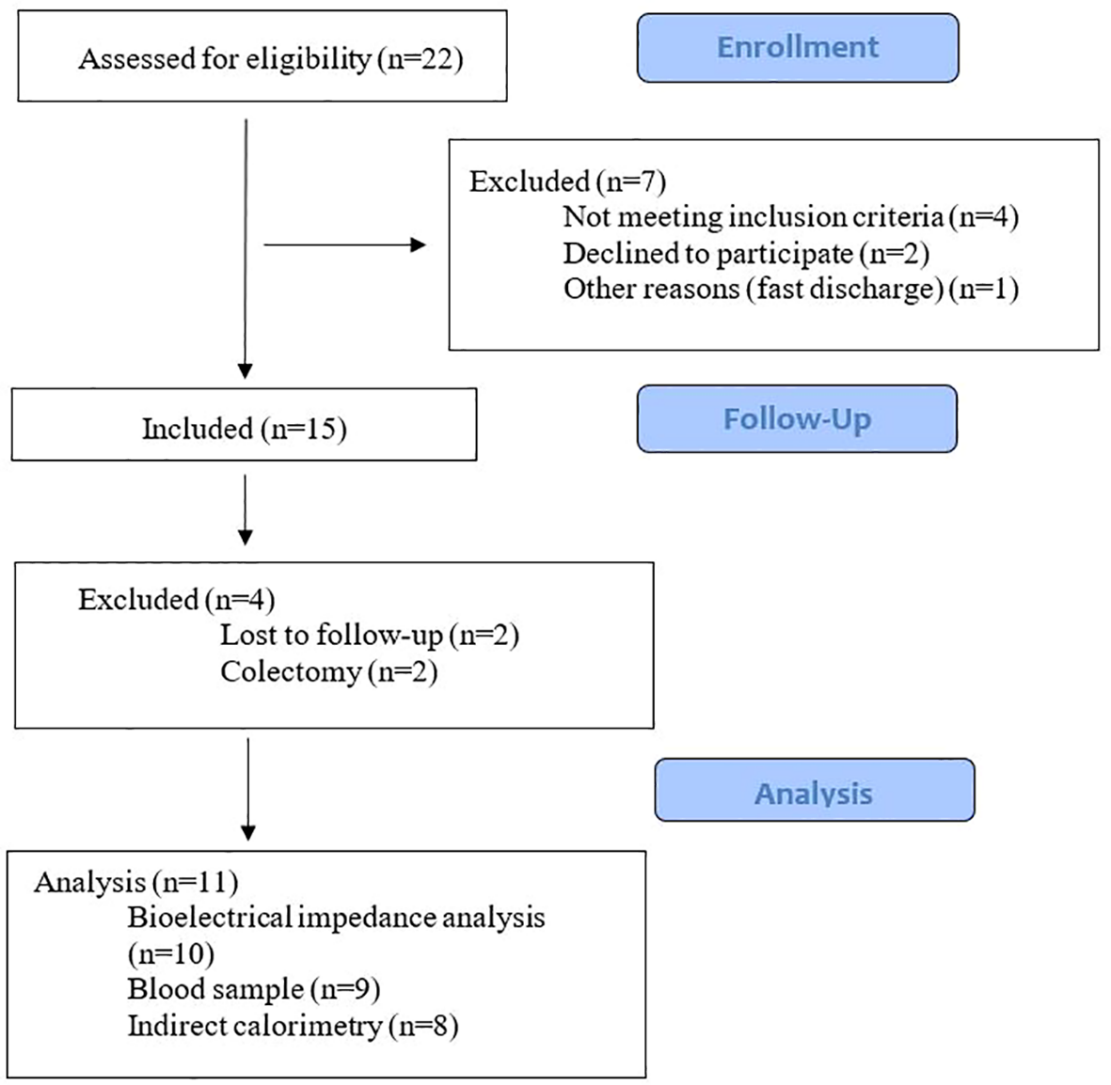
Study flowchart.

Fifteen out of 22 eligible patients with ASUC were enrolled in the study after providing written consent. During the study period, four patients were lost to follow-up due to colectomy (n=2) or lack of follow-up attendance (n=2). Eleven patients attended the follow-up visit (**Figure 1**). Their baseline characteristics are shown in **Table 1**. The median age across gender was 27 years [18; 76] and 11 (73%) of the participants were men. Eight patients (72%) were diagnosed with extensive UC. They had a median 1-year disease duration [0; 7]. The median CRP at admission was 37.6 [4; 154.7]. Further characteristics of the reference groups are presented in **Table 2**.

**Table 1:** Baseline characteristics of patients included in the present study (n=15).

Characteristics	Male (n=11)	Female (n=4)
Age (years)	24 [18; 76]]	35 [19; 63]
Body weight (kg)	80.5 [52.95; 100.3]	64.0 [57.6; 65.5]
Body Mass Index	24.0 [17.5; 33.2]	23.5 [21.7; 24.2]
Fat mass (%)	22.8 [6.2; 39.6]	26.4 [17.9; 33.3]
Skeletal muscle mass (%)	39.3 [25.5; 45.1]	33.3 [29.2; 40.0]
Years since diagnosis of UC	1 [0; 7]	2 [0; 6]
Bloody stools per day	8.5 [8; 15]	9 [4; 11]
CRP (mg/l)	52.9 [4; 173.3]	25.7 [13; 47]
SCCAI	10 [7; 13]	7,5 [6; 10]
Hemoglobin (mmol/l)	8.3 [6.1; 9.1]	7.65 [6.4; 81]
Temperature (Celsius)	37.6 [36.3; 39.6]	37.4 [36.8; 37.8]
UC classification, proctitis (*n*)	1	1
UC classification, left-sided (*n*)	2	1
UC classification, extensive (*n*)	8	2

CRP, C-reactive protein; SCCAI, Simple Clinical Colitis Activity Index; UC, ulcerative colitis.

Data are presented as median [range].

**Table 2:** Characteristics of reference groups.

Characteristics	Inpatients (without UC) (n=12)	Outpatients (with UC) (n=6)
Male	66.5%	66.5%
Age (years)	56.5 [44; 84]	35 [24; 45]
Body weight (kg)	74.4 [54.1; 119.8]	76.7 [63.1; 95.2]
Body Mass Index	25.4 [21.4; 37.4]	24.4 [20.4; 27.5]
Fat mass (%)	29.2 [22.4; 45.9]	21.2 [14.0; 34.2]
Skeletal muscle mass (%)	31.7 [22.6; 34.7]	38.2 [31.3; 34.2]
CRP (mg/l)	58.5 [28.6; 203]	<4.0 [0]
Steroid treatment	None	None
SCCAI	N/A	0 [0]
p glucose (mmol/l)	5.45 [4.8; 6.4]	4.9 [4.5; 5.7]
p insulin (mmol/l)	78.5 [27; 141]	33 [31; 75]
HOMA-IR	2.8 [0.8; 5.1]	2.0 [0.9; 2.8]

CRP, C-reactive protein; HOMA-IR, the Homeostatic Model Assessment for Insulin Resistance; p, plasma; SCCAI, Simple Clinical Colitis Activity Index; UC, ulcerative colitis.

Data are presented as median [range].

### 3.2 Insulin sensitivity

**Figure 2:**
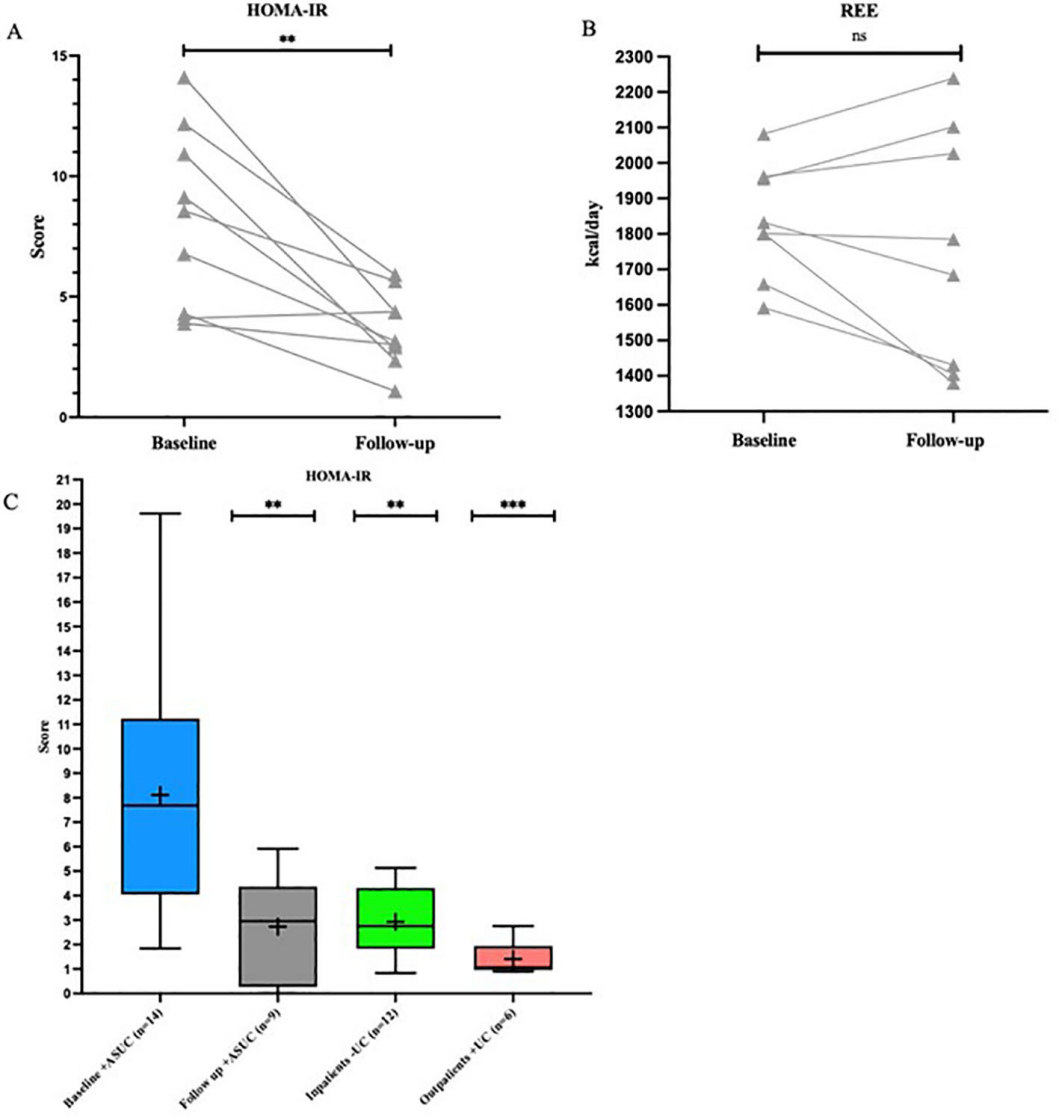
Before-after plot of HOMA-IR and REE and box plot for HOMA-IR. **(A, B)** Before-after plot illustrating the development of HOMA-IR and REE from baseline to follow-up. ** = p ≤ 0.01, ns = not significant. **(C)** HOMA-IR. Box plot comparing HOMA-IR between baseline and follow-up in the study group and the reference groups. ** = p ≤ 0.01 compared with group “Baseline +ASUC”, *** = p ≤ 0.001 compared with group “Baseline +ASUC”. ASUC, acute severe ulcerative colitis; HOMA-IR, Homeostatic Model Assessment for Insulin Resistance; REE, resting energy expenditure; UC, ulcerative colitis.

Median HOMA-IR was markedly elevated at inclusion, i.e. during hospitalization for ASUC and at steroid onset, and dropped statistically significantly from 8.6 [3.8; 14.1] at inclusion to 3.15 [1.08; 5.91] at follow-up (p=0.0036) (**Figure 2**). A reduction in HOMA-IR was observed in eight of nine patients.

Fasting plasma glucose decreased from a median 7.1 [6; 8.7] to 5.4 [4.8; 6.4] mmol/l (p=0.0039), whereas fasting plasma insulin decreased from 163 [85; 337] to 85 [35; 170] pmol/l within the same period (p=0.02) (**Table 3**).

**Table 3:** Anthropometric data, resting energy expenditure and insulin sensitivity markers at baseline and follow-up.

Parameter	Baseline	n	Follow-up	p-value
Body weight (kg)	76.2 [57.6; 100.3]	10	81.7 [59.7; 100.5]	0.2754
Skeletal muscle mass (%)	36.3 [28.5; 40.1]	10	36.4 [28; 42]	0.7695
Skeletal muscle mass(kg)	29.2 [18.5; 35.1]	10	28.5 [18.2; 34.3]	0.2324
Fat mass (%)	24.2 [16.6; 39.6]	10	23.8 [16.3; 40.3]	0.3750
Fat mass (kg)	20.1 [10.3; 33.7]	10	20.6 [11.4; 35.2]	0.2324
Phase angle (°)	5.4 [4.2; 6.6]	10	5.6 [4.4; 6.4]	0.2773
REE (kJ/day)	7,602 [6,664;8,715]	8	7,263 [5,777;9,372]	0.3125
REE (kJ/kg/day)	100 [78.3; 118.5]	8	92.9 [68.7; 112.2]	0.1094
RQ	0.9 [0.8; 1.0]	8	0.9 [0.8; 1.1]	0.6641
SCCAI	8 [6; 12]	11	0 [0; 2]	0.0010
CRP (mg/l)	37.6 [4; 154.7]	9	4.0 [0]	0.0078
p glucose (mmol/l)	7.1 [6; 8.7]	9	5.4 [4.8; 6.4]	0.0039
p insulin (pmol/l)	163 [85; 337]	9	85 [35; 170]	0.0156
HOMA-IR	8.6 [3.8; 14.1]	9	3.2 [1.1; 5.9]	0.0036

CRP, C-reactive protein; HOMA-IR, the Homeostatic Model Assessment for Insulin Resistance; p, plasma; REE, resting energy expenditure; RQ, respiratory quotient.

Data are presented as median [range].

In outpatients with UC in remission, a median HOMA-IR of 1.07 (0.89; 2.75) was observed, which was below the reference level. Inpatients with active inflammation but without UC had a median HOMA-IR of 2.75 (0.83; 5.13). Both reference groups showed a statistically significantly lower HOMA-IR than patients with ASUC at admission (**Figure 2**).

### 3.3 Indirect calorimetry and anthropometric measurements

At admission, the participants had a median REE of 7,602 [6,664; 8,715] kJ/day (1,816 kcal/day), corresponding to 100 [78.3; 118.5] kJ/kg/day (24.64 kcal/kg/day) (**Table 1**)]. A REE increase between baseline and follow-up was observed in three (38%) of eight patients, whereas a decrease in REE was observed in five (63%) of eight patients with a median of -330.7 [-1,762; 657.2] kJ/day (-79 [-421; 157] kcal/day) (**Figure 2**)]. No significant changes were observed in REE (kcal/day) and RQ between baseline and follow-up.

Seven out of ten patients gained weight from baseline to follow-up, with a median increase of 0.43 [-2.4; 8.35] kg. We observed no statistically significant changes in anthropometric measurements during the study period, including skeletal muscle mass (kg, %), fat mass (kg, %), and phase angle (°) (**Table 3**).

### 3.4 Correlation analyses

Correlation analyses of HOMA-IR and possible influencing variables, e.g., duration of steroid treatment, CRP (mg/L), age, and fat mass (kg), were performed for all outcomes. All analyses showed weak (r_s_ = 0,20-0,39) or very weak (r_s_ = 0,00-0,19) correlations (ns).

## 4 Discussion

In this small cohort study, we investigated the level of metabolic stress in patients admitted with ASUC. In essence, we found that the metabolic stress level, indicated mainly by insulin resistance, was higher among the admitted patients with ASUC compared to those from the reference groups. Our findings could indicate that there may exists a synergism between active inflammation and steroid treatment, with increased sensitivity during the admission period. It has not been possible to confirm these findings from other, similar studies. Thomsen et al. observed a HOMA-IR within the reference level before steroid treatment initiation (Solu-Medrol 80 mg/day) in patients admitted with ASUC (14). In the present study, HOMA-IR was assessed one or two days after steroid treatment initiation. These findings could support a vicious circle of inflammation and steroid treatment increasing metabolic stress.

Insulin resistance persisted in most patients 3 weeks after discharge and at clinical remission. A possible explanation for this may be that the participants on average still received relatively high doses of oral prednisolone at this point after discharge (35 mg/day). Doğan et al. observed a significantly elevated HOMA-IR in patients with UC in remission with and without receiving steroid treatment compared with healthy subjects (15). Our study could observe the same tendency in HOMA-IR.

Thorell and coworkers observed a normalization of insulin sensitivity after 20 days in patients undergoing elective surgery (16). Based on the results of this study, the degree of insulin resistance may be increased for a longer period in patients with ASUC than in patients undergoing elective surgery. However, repeated HOMA-IR measurements and an extension of the study period could have contributed with further knowledge.

It is well known that steroid treatment and accumulation of steroid treatment induces insulin resistance (17). Insulin resistance from steroid usage is closely linked to the risk of developing diabetes (10) and so is IBD to diabetes (18). In addition, cardiovascular disease is another major derivative morbidity caused by insulin resistance (19). Human clinical data suggest a six-fold increased risk of cardiovascular disease in patients with IBD (20). Insulin was not measured in the above-mentioned study. The study further adds to the theory that insulin resistance may be an important risk factor in IBD patients though this was not addressed. In yet another study, patients without diabetes with IBD were treated with anti-tumor necrosis factor alfa to examine insulin resistance with regards to insulin concentrations and HOMA-IR, showing a significantly improved insulin sensitivity compared with controls treated with aminosalicylates (21). Likewise, further data suggest a potential role of biological treatments used in maintenance and remission periods, such as TNF-α inhibitors, as a protective factor against nonalcoholic fatty liver disease in patients with IBD (22, 23).

This adds to the debate of how acute and chronic exacerbations in UC are best treated. Based on the known increased risk of glucocorticoid dose-dependent type 2 diabetes in IBD (24), it is increasingly evident that treatment with steroids in UC should be minimized.

Our study has important limitations. Firstly, the reduced sample size may have predisposed the study to a possible type II error. Secondly, the use of HOMA-IR for expressing insulin resistance carries some limitations. Fasting p-insulin may not reliably indicate insulin production as a significant amount is extracted by the liver during first passage and steady-state conditions are necessary for concentrations to reflect production. The gold standard for assessing insulin sensitivity is the hyperinsulinemia euglycemia clamp (HEC). This method was not applied in the present study due to its complexity. HOMA-IR is a simpler method than HEC and it is not invasive. Furthermore, a strong positive correlation between HOMA-IR and HEC was observed within other patient groups (25).

Based on our finding values of HOMA-IR in the two reference groups they seemed to be more or less insulin resistant, although with a lower median HOMA-IR compared to the patients with ASUC at baseline and follow-up. It can therefore be discussed, whether a third reference group in form of healthy, non-obese subjects should have been included. In a recent cohort study, 320 healthy participants was stratified into insulin sensitive and adiposity subgroups defined by BMI and HOMA-IR values (26). At baseline the subgroup with insulin-resistant non-obese subjects had a mean HOMA-IR and BMI of 3.35 and 26.4, respectively. Thus the HOMA-IR and BMI was higher compared to the reference groups in our study. In addition, this could indicate that despite we found a mean difference between our inpatients and outpatients, it would not influent the results of their HOMA-IR. Lastly, the HOMA-IR analysis was performed after the initiation of the steroid therapy, which can be a potential confounding factor for the conclusions of this study.

## 5 Conclusions

In conclusion, the present study adds new explorative data to the existing literature documenting insulin resistance during acute inflammation with steroid treatment. Additionally, the findings of the study suggest that patients with acute severe ulcerative colitis should be closely monitored for insulin resistance. To support the findings of the present study, further research is warranted.

## Data Availability

The raw data supporting the conclusions of this article will be made available by the authors, without undue reservation.
